# Application of Big Data and Artificial Intelligence technologies to dementia prevention research: an opportunity for low-and-middle-income countries

**DOI:** 10.7189/jogh.09.020322

**Published:** 2019-12

**Authors:** Samuel O Danso, Graciela Muniz-Terrera, Saturnino Luz, Craig Ritchie, on behalf of the Global Dementia Prevention Program (GloDePP)

**Affiliations:** 1 Edinburgh Dementia Prevention, Centre for Clinical Brain Sciences, University of Edinburgh, Edinburgh, Scotland, UK; 2 Usher Institute of Population Health Sciences and Informatics, Edinburgh Medical School, Molecular, Genetic and Population Health Sciences, University of Edinburgh, Edinburgh, Scotland, UK

It is estimated that about 66% of the number of people with dementia globally live in regions within the low-and-middle-income countries (LMIC). The estimates further suggest that about 1 million additional cases are recorded annually in these LMICs, with approximately 60-70% of the dementia cases being due to Alzheimer disease, AD [1]. Despite this high incidence of AD, treatment options are currently limited. This is notwithstanding the fact that numerous clinical trials have taken place, are still on going or planned [2]. However, the rate of failure of clinical trials and predictions based on available results from randomised control trials suggest that an immediate breakthrough to obtaining treatment drugs for AD is unlikely [3].

While the symptoms of dementia appear to be well known, including decline in activities of daily living and social functioning, the exact cause remains unknown [4]. However, there is strong evidence based on several epidemiological studies to suggest that complex interactions exist between exposures such as adverse environmental factors and lifestyle choices and how these contribute to the risk and timing of dementia onset [5,6]. A comprehensive account of the various risk factors that interact to influence the risk profile and the timing of dementia onset is provided in the recently published Lancet Commission report [4]. The report furthermore suggests that the effect of these risk factors in terms of their contribution to dementia are beginning to be understood through complex modelling. These risk factors can be categorised into modifiable (such as lifestyle) and non-modifiable (such as genetics). The ability to alter the modifiable risk factors has been demonstrated theoretically to have influence on the onset of dementia. For example, it may be possible to reduce dementia prevalence by 50% if the onset was to be delayed by 5 years based on population-attributable risk estimation [7].

In the absence of strong epidemiological data from LMIC, evidence from numerous epidemiological studies carried out in high-income-countries (HIC) point to the fact that prevention is vitally important to reducing the dementia burden. These findings may be extended to LMIC and this article discusses these opportunities and how they can be applied.

**Figure:**
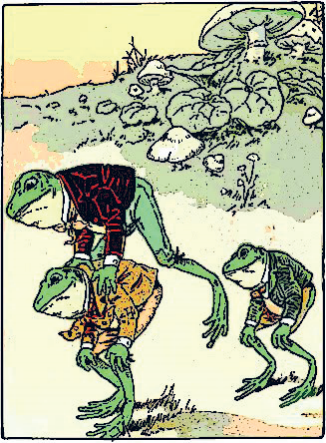
Photo: LMICs leapfrogging with collaborative support and lessons leaned from HICs to tackle dementia (photo taken from wpclipart collections: used under the terms and conditions).

## MODIFIABLE RISK FACTORS

It has now been established that the onset of dementia begins much earlier in life than had previously been assumed [5]. This is further supported by evidence from clinicopathologic studies, which suggest that AD lesions develop much earlier in specific regions of the brain which meet neuropathological AD diagnosis criteria even in the absence of dementia [8]. Meanwhile, numerous studies have also reported the effect of modifiable risk factors on the structural integrity of the brain. Examples include a 20-year longitudinal study, which found that cardiovascular risk factors such as hypertension, BMI from midlife to late life also increased the risk of severe white matter lesion in the brain [9]. A recent study also found a correlation between hypertension and reduction in brain reserve[10], and therefore adopting a healthy lifestyle may reduce the chances of hypertension which in turn also delay the onset of AD . Similar trends have been observed in nutrition studies [11]. Lifestyle factors such as physical activity through longitudinal observational studies have similarly been demonstrated to have improved cognitive reserves and reduction in dementia risk[12]. Furthermore, studies on lifestyle have also found smoking and excessive alcohol intake to have significant association with cognition and dementia[13], with findings from education also showing some mixed effect on cognition and cognitive preservations [14]. Establishing norms for LMIC populations for these risk factors and developing monitoring mechanisms capable of providing early warning signs is an avenue to the development of strategies that can focus on early interventions.

## DEMENTIA EARLY INTERVENTION THROUGH BIG DATA AND AI IN LMIC

HIC continue to explore advanced techniques in neuroimaging as wells as neuropsychological and other data sources to effectively monitor brain health to detect early onset of dementia [15,16]. Recent advances in Big Data and AI technologies coupled that with the ever-increasing speed in data generation has seen exponential growth in research and development of these technologies within the context of dementia prevention. Researchers in HIC have already taken advantage of Big Data technologies and have developed data driven approaches to dementia prevention initiatives across various HICs and are able to process and manage these data with high throughput [17]. Current examples of such initiative include the ongoing European Prevention of Alzheimer Disease, EPAD [18] and PREVENT research programme [5]. These projects aim to generate not only large and high quality, but also phenotypically deep, data sets and are employing state-of-the-art Big Data technologies to process and make these data available through secured analytics environment for hypothesis testing.

Similar trends have also been observed in the analytics space where AI including sophisticated Machine Learning (ML) algorithms and frameworks such as deep learning continue to be developed and improved. A systematic review by Pellegrini et al.[19] found over 110 publications on various initiatives where ML approaches have been employed to develop prediction models for cognitive impairment and AD using neuroimaging data. These research efforts are being extended to other data sources and domains such as linguistic analysis of text messages; speech analysis [20,21]; and also through the human eyes using retinal imaging [22] with promising results.

While ML models based on neuroimaging data may not be practical in LMIC due to the huge cost associated with them, having alternative low cost approaches to acquiring modifiable risk factors data and analysing these data sources to help monitor populations brain health for the purposes of screening for dementia risks would be a useful epidemiological tool. For example, wearable devices and smartphones are now able to acquire real time data on daily activities, conversations through text messages and speech. The proliferation of the internet and smartphone usage in LMIC presents great opportunities for collecting these types of data using these devices. Big Data technologies allow efficient integration of data that come from a variety of sources and in different types and formats scale [23]. Harmonising these sources of data provides an excellent opportunity to develop ML methods and prediction models. AI and machine learning technologies are also revolutionising the approaches to the analysis of large volumes of data in real time, with potential benefits to dementia research, including the ability to learn patterns from large data sets where use of traditional statistical methods may not be possible [24]. Additionally, the efficiencies and consistency in the use of AI–based methods may result in reduced cost and less human errors in decision-making.

## REGULATORY ISSUES

It is an undeniable fact that Big Data and AI have potential ethical challenges such as data privacy and respect for human rights. This has led to a shift in focus and a new branch of ethics known as data ethics, which involves the study of moral issues around data, algorithms and practices [25]. Regardless of the place of application, these ethical issues remain universal and applicable in LMIC. Nevertheless, regulatory frameworks such as the recently launched General Data Protection Regulations in May 2018 to regulate the use of personal data across Europe [26] and practises employed in other HIC such as the consent approach where study participants consent to researchers keeping their personal data centrally for the purposed of data linkage but these are not transferred to the data users. Another approach is the de-identification method where personal identifiable data are removed at the point of data collection before further processing and transmission as described in [23]. These approaches can serve as good examples to follow in LMIC.

## CONCLUSION

This article discussed opportunities for development of early intervention strategies to address the burden of dementia in LMIC through the application of Big Data and AI. The potential efficiency and cost-effectiveness of this approach suggests that window of opportunity now exists for researchers in LMIC to collaborate with colleagues in HIC in this area. LMIC can employ a leapfrog approach to adapt in order to benefit from these Big Data and AI technologies, which have already been tried and tested in HIC. With this approach, the emerging success stories in HIC can also be replicated in LMIC.
